# Interval to vascularization development in cirrhotic precursor nodules in patients with hepatitis B and C virus co-infections

**DOI:** 10.1371/journal.pone.0178841

**Published:** 2017-06-07

**Authors:** Nai-Chi Chiu, Chien-Wei Su, Chien-An Liu, Yi-Hsiang Huang, Yi-You Chiou

**Affiliations:** 1 Department of Radiology, Taipei Veterans General Hospital, Taipei, Taiwan; 2 School of Medicine, National Yang-Ming University, Taipei, Taiwan; 3 Division of Gastroenterology and Hepatology, Department of Medicine, Taipei Veterans General Hospital, Taipei, Taiwan; Chang Gung Memorial Hospital Kaohsiung Branch, TAIWAN

## Abstract

With the widespread use of gadoxetic acid-enhanced magnetic resonance imaging, liver nodules appearing as hypovascular in the arterial phase and hypointense in the hepatobiliary phase, defined as hypovascular hypointense nodules, are increasingly detected in patients with cirrhosis and are considered precursor nodules. We sought to evaluate the interval to vascularization development in hepatitis C virus/hepatitis B virus co-infected-associated precursor nodules (BC-HHN group) compared with that in hepatitis C virus mono-infected-associated precursor nodules (C-HHN group) in the hepatobiliary phase of gadoxetic acid-enhanced magnetic resonance imaging. The interval to vascularization development was estimated by the Kaplan-Meier method and compared using the Cox proportional hazards model. The mean intervals to vascularization development in the BC-HHN and C-HHN groups were 272.9±31.1 and 603.8±47.6 days, respectively (*p*<0.001). The cumulative vascularization development incidence at 6, 12, and 18 months was 44.9%, 73.5%, and 91.8%, respectively, in the BC-HHN group and 16.9%, 39.0%, and 55.8%, respectively, in the C-HHN group (*p*<0.001). The multivariate analysis showed that the presence of hepatitis B virus co-infection (hazard ratio: 1.819; 95% confidence interval: 1.222–2.707; *p* = 0.003) and male sex (hazard ratio: 1.753; 95% confidence interval: 1.029–2.985; *p* = 0.039) were predictors of vascularization development. More than half of the hypovascular hypointense nodules showed high-signal changes on T2-weighted imaging, and almost half of them showed restricted diffusion on diffusion-weighted images, but these did not predict vascularization development. In a hepatitis C virus- and hepatitis B virus-endemic area, such as Taiwan, precursor nodules in the BC-HHN group tended to have shorter intervals to vascularization development, especially in male patients.

## Introduction

Most patients with hepatitis C experience chronic hepatitis C virus (HCV) mono-infection. However, in areas where hepatitis B virus (HBV) infection is endemic, such as Southeast Asia, the Far East, and southern Europe, a substantial number of patients are infected with both HBV and HCV. The prevalence of chronic HBV and HCV infections in the general population in Taiwan is approximately 0.95–2.6% and 13.7–20%, respectively [1–3]. The estimated prevalence of HBV and HCV co-infections is about 0.26–2.4% in Taiwan [4, 5]. In patients with HBV and HCV co-infections, clinical presentations and disease outcomes are usually more severe than in patients with mono-infections [6–8]. Most studies have provided substantial evidence to support that HBV and HCV co-infections increase the risk of fulminant hepatic failure, the progression of liver disease, and the development of hepatocellular carcinoma (HCC) [4, 9].

HCC has been proven to develop by multi-step carcinogenesis from a dysplastic nodule to early HCC and finally to hypervascular HCC [10]. The inflow of nodules changes from a primarily portal supply to an exclusively arterial supply during the multi-step hepatocarcinogenesis [11]. Dysplastic nodules show decreased arterial enhancement compared with that in the surrounding liver parenchyma in the arterial phase. On the other hand, moderately differentiated HCC shows clearly increased arterial enhancement. Thus, it is important to monitor the evolution of vascularization development, from precursor nodules to hypervascular HCC.

Gadoxetic acid is a dual-function extracellular contrast agent that has been proven to help detect early HCC; approximately 50% of an injected dose is taken up by hepatocytes and excreted in bile [12]. With the widespread use of gadoxetic acid-enhanced magnetic resonance imaging (MRI), liver nodules appearing as hypovascular in the arterial phase and hypointense in the hepatobiliary phase (HBP), defined as hypovascular hypointense nodules (HHNs), are increasingly detected in patients with cirrhosis [13]. HHNs detected during the HBP on gadoxetic acid-enhanced MRI can be considered as precursor nodules, including regeneration nodules, low-grade dysplastic nodules, high-grade dysplastic nodules, or even early HCC. It is important to treat a precursor nodule as HCC when HHN vascularization is detected early [14]. Previous studies have reported that the cumulative vascularization development in HHNs ranged from 10% to 43.5% over a 1-year follow-up period [15–17]. Moreover, recent studies have revealed an association between the development of HHN vascularization and the initial nodule size, hyperintensity on T1- and T2-weighted images, intra-nodular fat, and diffusion restriction [15, 18–20].

As HBV and HCV co-infections may result in more severe liver cirrhosis, we hypothesized that HHNs in such cases might have a shorter interval to subsequent vascularization development than would those resulting from HCV mono-infection. To test this hypothesis, this study aimed to compare the HHN vascularization development in patients with HCV mono-infection and HBV and HCV co-infections.

## Materials and methods

### Subjects

**Fig 1:**
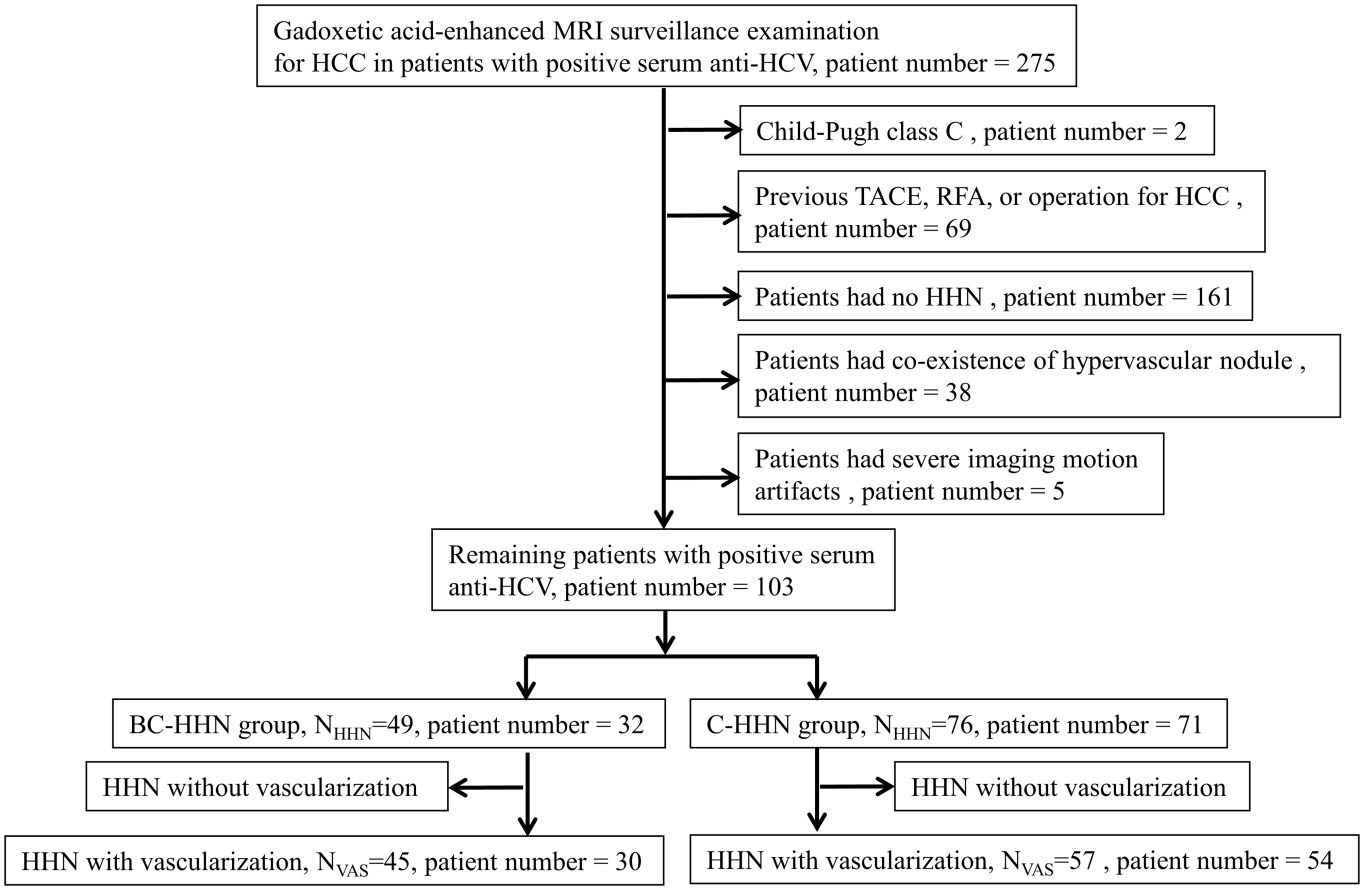
Flow chart for the selection of the study population. HCC, hepatocellular carcinoma; HHN, hypovascular hypointense nodule.

Fig 1 shows the flow chart for the selection of the study population. The study involved a retrospective review of 103 HCV-infected patients from 2009 to 2014 who underwent gadoxetic acid-enhanced MRI because of suspicious nodular lesions detected by an ultrasonography surveillance examination for HCC. HCV-infected patients were defined as those with positive serum antibodies against HCV (anti-HCV). As the co-existence of hypervascular nodule was assumed to be a risk factor for subsequent HHN vascularization, we excluded those patients who had a previous treatment of HCC and those with co-existence of hypervascular nodule. Because of insufficient enhancement during the HBP on gadoxetic acid-enhanced MRI, we excluded those patients who were identified with Child-Pugh class C disease. Transient respiratory motion artifacts in gadoxetic acid-enhanced MRI may limit optimal arterial phase imaging, so we excluded those with severe imaging motion artifacts. It was approved by our Institutional Review Board (approval number 2014-09-001BC, S1 Fig), and the need to obtain informed patient consent was waived. Patient information was de-identified before the initiation of this study.

We further divided the patients into two groups by the status of serum HBV surface antigen (HBsAg). Thirty-two HCV-infected patients positive for HBsAg were assigned to the HBV and HCV co-infection group; these 32 patients had 49 HHNs (BC-HHN group). Seventy-one HCV patients negative for serum HBsAg were assigned to the HCV mono-infection group; these 71 HCV patients had 76 HHNs (C-HHN group). All nodules were followed until vascularization development or at least 730 days with follow-up contrast-enhanced dynamic MRI examinations at 3-month intervals until vascularization development was detected. Vascularization was defined as enhancement of the HHN observed during the arterial phase on follow-up MRI. We obtained the patients’ baseline clinical characteristics by reviewing all available medical records in order to determine their associations with the interval to vascularization development in HHNs.

Patient characteristics collected were age, sex, serum albumin levels, total bilirubin, platelet, serum aspartate aminotransferase (AST), alanine aminotransferase (ALT) levels, the prothrombin time-international normalized ratio (PT-INR), and alpha-fetoprotein (AFP). Laboratory data obtained within 1 month of the initial MRI examination were recorded. Serum HBsAg and anti-HCV were tested by radio-immunoassay (Abbott Laboratories, North Chicago, IL) and second-generation enzyme immunoassay (Abbott). Serum biochemistries were measured using a Roche/Hitachi Modular Analytics System (Roche Diagnostics GmbH, Mannheim, Germany). The serum AFP level was tested using a radio-immunoassay kit (Serono Diagnostic SA, Coinsin/VD, Switzerland).

### Imaging techniques

All initial MRI examinations were performed using a 1.5-Tesla MRI system (Signa EXCITE HD; GE Medical Systems). We obtained two-dimensional gradient-echo (GRE) T1-weighted images with dual-echo acquisition (repetition time/echo times [TR/TEs], 180 ms/2.2–4.5 ms; flip angle, 80°; matrix, 256 × 224; field of view [FOV], 350 × 320 mm; slice thickness, 8 mm; fat-suppressed fast spin-echo [FSE] T2-weighted images [TR/TE], 3378–4800/68–76 ms; matrix, 288 × 224; FOV, 350 × 320 mm, slice thickness, 6 mm). Three-dimensional dynamic fat-suppressed GRE T1-weighted images (TR/TE, 4.3/2.1 ms; flip angle, 12°; matrix, 288 × 192; FOV, 350 × 320 mm; slice thickness, 3 mm) were taken before and at 30 s (arterial phase), 60 s (portal phase), 90 s (equilibrium phase), and 20 min (HBP) after a bolus injection of gadoxetic acid (Primovist; Bayer Schering Pharma, 0.025 mmol/kg) administered at a rate of 1 mL/s with a 20-mL saline flush through the antecubital line (20–22 G). All images were obtained in the transverse plane.

### Image analysis

**Fig 2:**
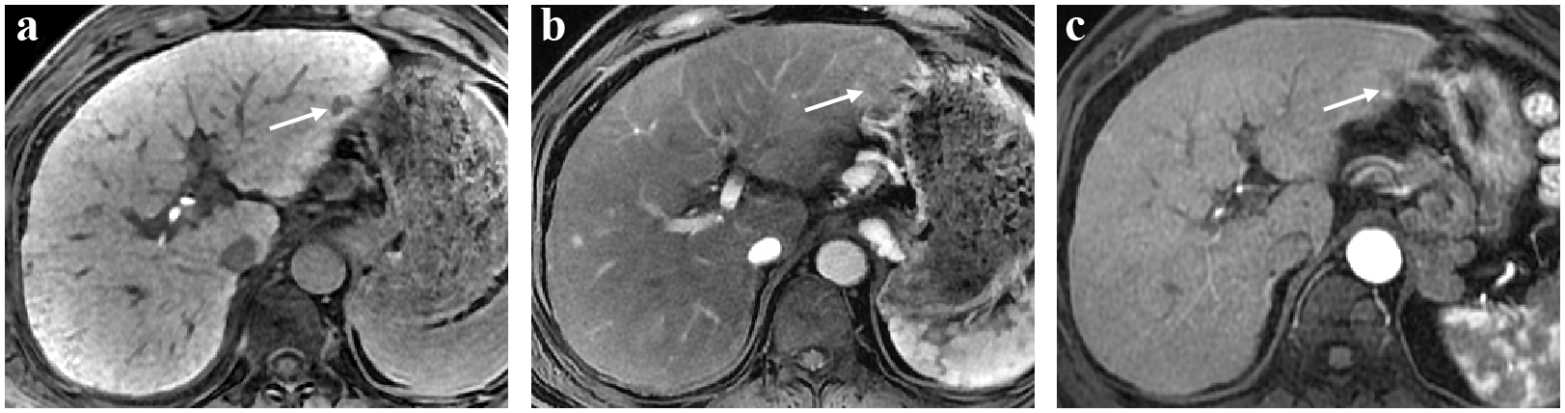
(A) Hepatobliiary phase (HBP) of gadoxetic acid-enhanced MRI, showed a hypointense nodule (arrow), which was considered as a liver nodule that showed a signal that was lower than that in the surrounding normal liver parenchyma. (B) Arterial phase of gadoxetic acid-enhanced MRI, showed a hypovascular nodule (arrow), which was defined as a liver nodule showing no evidence of arterial phase enhancement compared to that in the adjacent normal liver parenchyma. (C) Arterial phase of the follow-up MRI, showed vascularization development, which was defined as enhancement of the HHN.

All images were retrospectively interpreted in consensus by two experienced radiologists. All images were evaluated by using a picture archiving and communication system, with an adjustment of the optimal window setting in each case. A hypointense nodule was considered as a liver nodule that showed a signal that was lower than that in the surrounding normal liver parenchyma during the HBP of gadoxetic acid-enhanced MRI (Fig 2A, arrow). A hypovascular nodule was defined as a liver nodule showing no evidence of arterial phase enhancement compared to that in the adjacent normal liver parenchyma (Fig 2B, arrow). Vascularization development was defined as enhancement of the HHN observed during the arterial phase on follow-up MRI (Fig 2C, arrow). The initial HHN size was measured at its greatest diameter on the HBP image. The signal intensity of the HHN on T2-weighted imaging was classified as “high” or “isointense” as compared to that in the liver parenchyma. On T1-weighted imaging, the HHN was classified as “high” or “isointense” as compared to that in the liver parenchyma. *B* values of 0 and 800 sec/mm^2^ were used to generate the apparent diffusion coefficient map. The nodule volume doubling time (NVDT) was calculated as follows: NVDT = *T* × log2/ [3 × log(*D*_*2*_/*D*_*1*_)], where *T* is the time interval between two measurements, *D*_*2*_ and *D*_*1*_ denote the maximal diameter of the nodule at the initial and last MRI, respectively.

### Statistical analysis

The clinical characteristics of both groups were compared by using a Mann-Whitney U test for continuous values and Pearson’s chi-squared analysis or Fisher’s exact test for proportions. Continuous variables were presented as medians and ranges. The interval to vascularization development was calculated from the date of acquisition of the initial gadoxetic acid-enhanced MRI to the date of acquisition of the final follow-up MRI showing vascularization development. The cumulative vascularization incidence was estimated by the Kaplan-Meier method. A univariate Cox regression analysis of the clustered data was used to test for the association between the baseline characteristics and vascularization development. A multivariate analysis conducted by using a Cox proportional hazards model for the clustered data was performed to identify predictive variables while adjusting for the other characteristics. All variables were included in the full model, and the parameter estimates for this full model are provided. Hazard ratios (HRs) and the corresponding 95% confidence intervals (CIs) were reported. Variables with statistical significance (*p*<0.05) or proximate to it (*p*<0.1) in the univariate analysis were included in the multivariate analysis via a forward stepwise Cox regression model. A two-tailed *p*<0.05 was considered statistically significant. Correlation between nodule volume doubling time and interval to vascularization development was obtained using Spearman’s correlation coefficient for statistical analysis. All statistical analyses were performed using IBM SPSS Statistics for Windows, version 21.0 (IBM Corp., Armonk, NY, USA).

## Results

### Comparison of clinical demographics for BC-HHN and C-HHN

The main demographics and clinical data of the study population are shown in Table 1 (S1 Text). There was no significant difference in age distribution between the two groups (69 [56–74.5] vs. 65 [59–73] years, respectively). There was a trend toward male predominance in both groups. The serum albumin, total bilirubin, platelet, AST, ALT, BUN, creatinine levels, the PT-INR, and AFP were not significantly different between groups.

**Table 1:** Comparison of the demographic data of BC-HHN and C-HHN.

Parameter	BC-HHN (N_HHN_ = 49)	C-HHN (N_HHN_ = 76)	P^a^
• **Patient demographics**			
Age (y/o).^b^	69.0; 56.0–74.5	65.0; 59.0–73.0	0.421
Sex (M/F) (%)	31/18 (63.3/36.7)	40/36 (52.6/47.4)	0.324
• **Serum biochemistry and liver function tests**^b^			
Albumin (g/dL).^c^	4.0; 3.8–4.3	4.0; 3.85–4.3	0.769
Total bilirubin (mg/dL).^c^	0.84; 0.66–0.93	0.90;0.80–1.05	0.078
Platelet (mm^3^). ^c^	144,000; 111,500–190,000	138,000; 116,000–173,000	0.608
ALT (U/L). ^c^	41.0; 26.5–72.5	45.0; 32.0–69.0	0.657
AST (U/L).^c^	41.0; 31.5–56	44.5; 36.0–71.8	0.768
Creatinine (mg/dL).^c^	0.87; 0.73–1.06	0.86; 0.74–1.03	0.880
PT-INR. ^c^	1.07; 1.03–1.15	1.07; 1.03–1.11	0.829
AFP (ng/mL).^c^	9.98; 3.37–43.89	4.57; 2.29–13.00	0.065
• **HHN factors**.^b^			
HHN size (mm)	10; 9–12	12; 10–13	0.089
T2WI high-signal changes	27/49 (55.1%)	40/76 (52.6%)	0.788
T1WI high-signal changes	6/49 (12.2%)	10/76 (13.2%)	0.881
Restricted diffusion on DWI	25/49 (51.0%)	37/76 (48.7%)	0.799

^a^P: comparison between BC-HHN and C-HHN.

^b^Median; 25 and 75 percentiles.

^c^There were missing data at the time of Gadoxetic acid-enhanced MRI. ALT: alanine aminotrasferase. AST: aspartate aminotransferase. PT-INR: prothrombin time-international normalized ration. AFP: alpha-fetoprotein. HHN: hypovascular hypointense nodule.

### Comparison of HHN imaging factors for BC-HHN and C-HHN

The mean number of HHNs per patient was significant higher in the BC-HHN group (1.53 vs. 1.07, *p* = 0.042). The median size of HHNs in the BC-HHN group was not significantly different from that in the C-HHN group (10 vs. 12 mm, *p* = 0.089). However, HHNs in the BC-HHN group tended to be smaller than those in the C-HHN group. More than half of the HHNs showed high-signal changes on T2-weighted imaging, and almost half of the HHNs showed restricted diffusion on diffusion-weighted images, but no significant difference between the two groups was observed. Some of the HHNs showed high-signal changes on T1-weighted imaging, but no significant difference between the two groups was observed.

### Factors associated with vascularization development

**Fig 3:**
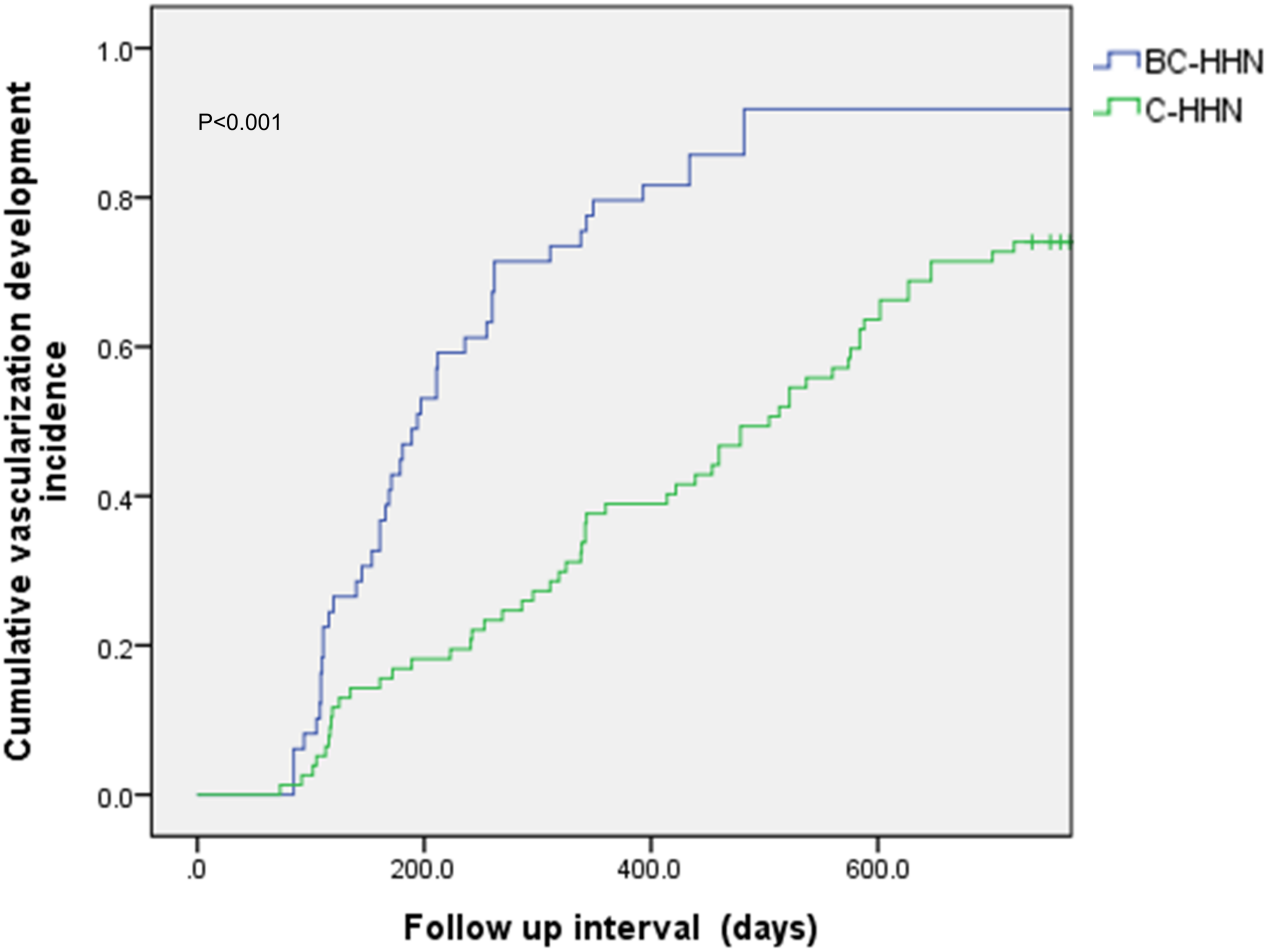
Cumulative incidence of vascularization development.

All nodules were followed until vascularization development or at least 730 days. During the medium and interquartile range (IQR) follow-up of days (339.00, 167.50–595.00), 45 of 49 BC-HHNs (91.8%) and 57 of 76 C-HHNs (75%) had evidence of vascularization development. Stratified by viral etiology, the medium and IQR follow-up of days was 194.00, 118.00–340.50 for BC-HHN group and 491.50, 273.25–732.00 for C-HHN group, respectively. In the BC-HHN and C-HHN groups, the mean interval to vascularization development was 272.9 ± 31.1 and 603.8 ± 47.6 days, respectively (*p*<0.001). The cumulative vascularization development incidence at 6, 12, and 18 months was 44.9%, 73.5%, and 91.8%, respectively, in the BC-HHN group, and 16.9%, 39.0%, and 55.8% in the C-HHN group (Fig 3) (*p*<0.001). In the BC-HHN group, 9 patients received TACE treatment and 1 patient received RFA treatment for the vascularized HHNs. In the C-HHN group, 12 patients received TACE treatment and 2 patient received RFA treatment for the vascularized HHNs. The multivariate analysis showed that the presence of HBV co-infection and male sex were the significant predictors of vascularization development (Table 2).

**Table 2:** Multivariate analysis of predict factors for vascularization development in BC-HHN or C-HHN (N_VAS_ = 102).

Univariate analysis				Multivariate analysis		
Variable	Hazard ratio	95% CI	P^a^	Hazard ratio	95% CI	P^a^
HBV co-infections	1.616	1.320–1.978	<0.001	1.819	1.222–2.707	**0.003**
Sex (M/F) (%)	1.274	1.064–1.432	0.031	1.753	1.029–2.985	**0.039**
Age (y/o)	0.996	0.977–1.016	0.710			
Albumin (g/dL).^c^	0.622	0.338–1.143	0.126			
Total Bilirubin.^c^	0.731	0.353–1.515	0.399			
ALT (U/L).^c^	0.990	0.982–0.998	0.020	0.998	0.961–1.036	0.927
AST (U/L). ^c^	0.992	0.984–1.001	0.078			
Creatinine (mg/dL). ^c^	1.369	1.010–1.856	0.043	1.649	0.912–2.979	0.098
PT-INR. ^c^	11.594	0.409–328.423	0.151			
Platelet	0.835	0.608–1.148	0.267			
AFP	1.085	0.834–1.412	0.544			
HHN size (mm)	0.998	0.922–1.079	0.952			
T2WI high-signal changes (%)	1.048	0.862–1.274	0.638			
T1WI high-signal changes (%)	0.936	0.706–1.242	0.647			
Restricted diffusion on DWI (%)	1.166	0.960–1.416	0.121			

^a^P: comparison between BC-HHN and C-HHN.

^b^Median; 25 and 75 percentiles.

^c^There were missing data at the time of Gadoxetic acid-enhanced MRI. CI: confidence interval. ALT: alanine aminotrasferase. AST: aspartate aminotransferase. PT-INR: prothrombin time-international normalized ration. AFP: alpha-fetoprotein. HHN: hypovascular hypointense nodule.

### Factors associated with vascularization development stratified by sex

**Fig 4:**
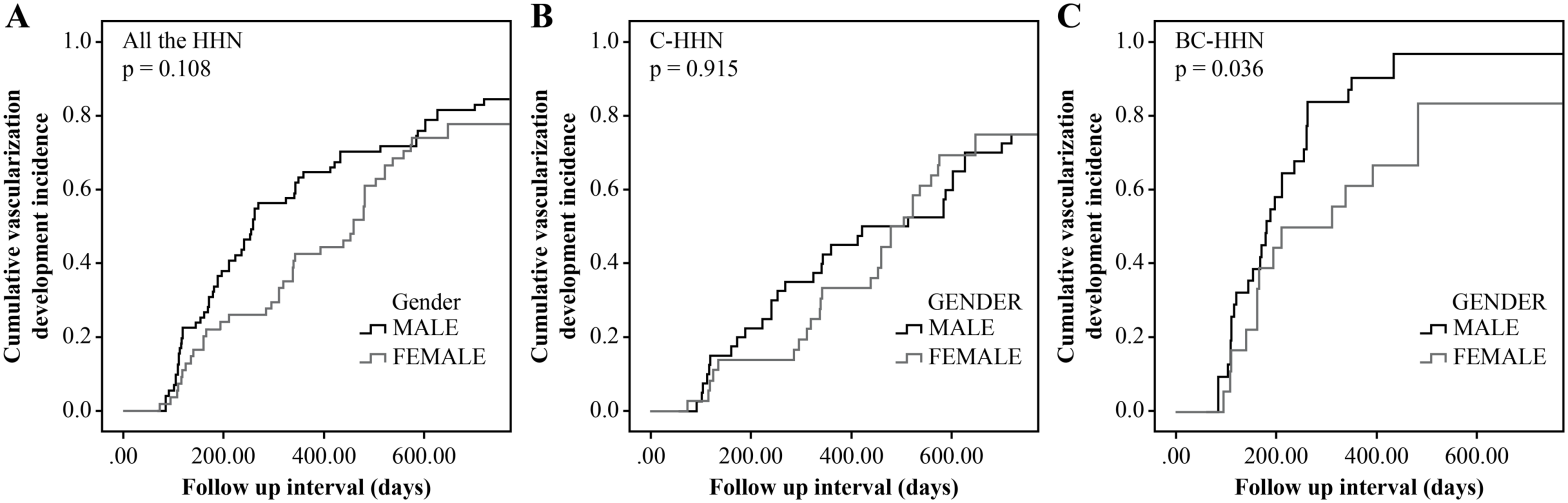
Comparison of cumulative incidence of vascularization development between sex stratified by groups. (A) all the HHN; (B) C-HHN; (C): BC-HHN.

**Fig 5:**
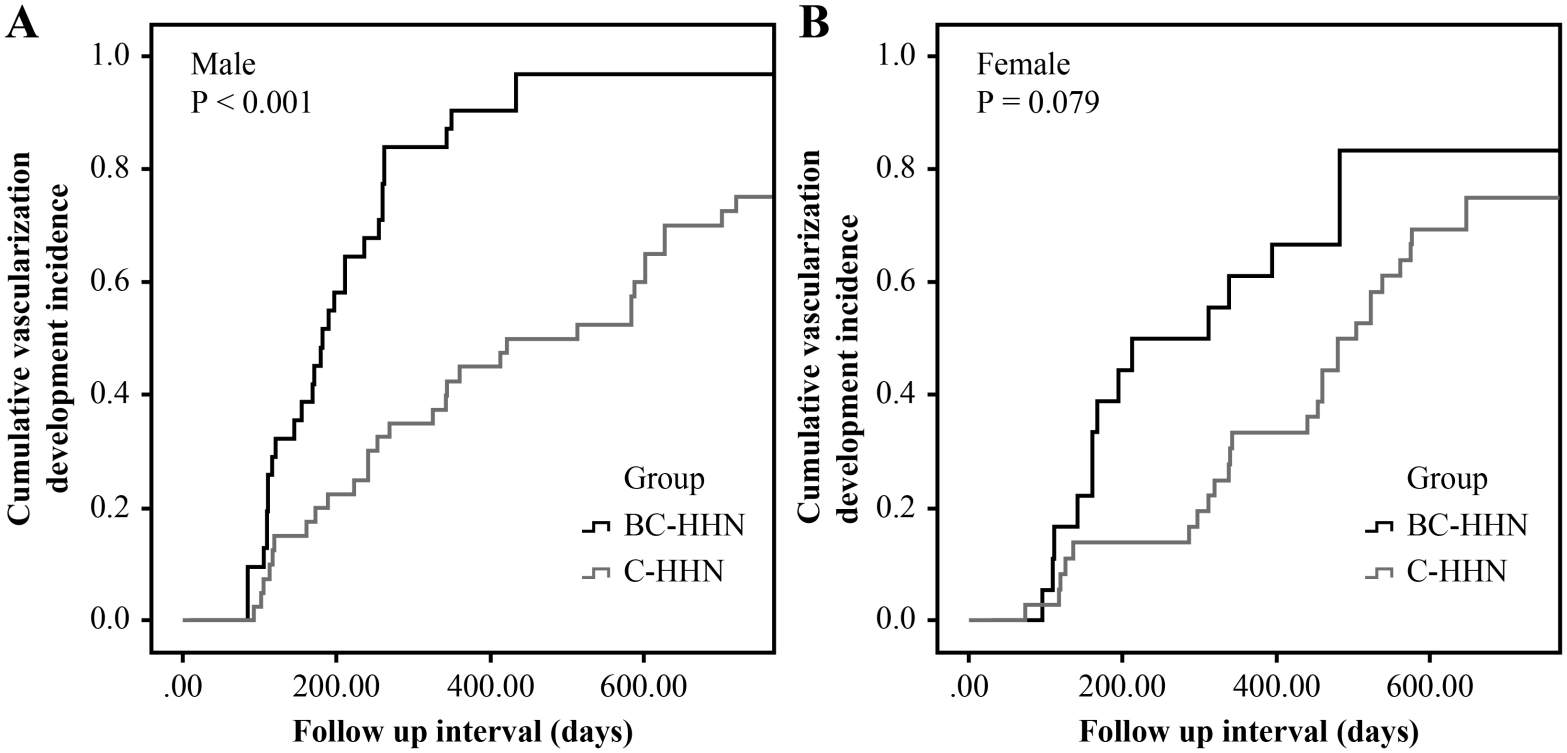
Comparison of cumulative incidence of vascularization development between groups stratified by sex. (A) male; (B) female.

The cumulative vascularization development incidence of BC-HHN in male patients was significantly higher than that of BC-HHN in female patients (*p* = 0.036) (Fig 4C). The cumulative vascularization development incidence by sex was not significantly different in C-HHN or for HHNs overall (Fig 4A and 4B). The cumulative vascularization development incidence of BC-HHN in male patients was significantly higher than that of C-HHN in male patients (*p*<0.001) (Fig 5A). The cumulative vascularization development incidence of BC-HHN in female patients was not significantly different from that of C-HHN in female patients (*p* = 0.079) (Fig 5B). We analyzed the effect of male sex on the cumulative vascularization development incidence separately for the BC-HHN and C-HHN groups. In the C-HHN group, male sex was not a predictor of vascularization development (HR: 1.014; 95% CI: 0.781–1.317; *p* = 0.92), but it was in the BC-HHN group (HR: 1.054; 95% confidence interval: 1.015–1.094; *p* = 0.006). Thus, male patients with HBV co-infection had the highest cumulative vascularization development incidence.

### Factors associated with vascularization development stratified by HHN size

In BC-HHN group, the medium and standard deviation of nodule size was 10.0 ± 2.4mm in male patients and 10.5 ± 2.1 mm in female patients (p = 0.66). By univariate analyses, HHN size was not a predictor of the cumulative incidence of vascularization development. Then, we examined whether HHN size was a predictor for vascularization development in the BC-HHN and C-HHN groups separately. There were no significant differences in the cumulative incidence of vascularization development between HHN size (≤8 mm, >8 mm) in BC-HHN, C-HHN, or HHN overall.

### Nodule volume doubling time analysis

Thirty-one nodules in BC-HHN group with vascularization (N_VAS_ = 45) and 25 nodules in C-HHN group with vascularization (N_VAS_ = 57) were evaluated. The nodule volume doubling time in BC-HHN was significant less than that in C-HHN (226.8 ± 30.5 days v.s 575.3 ± 99.3 days, *p*< 0.001). There was significant positive correlation between nodule volume doubling time and interval to vascularization development (*r* = .881**, *p*<0.001). There was significant negative correlation between nodule volume doubling time and vascularization development (*r* = -.390**, *p* = 0.001).

## Discussion

In the present study, the overall 6- and 12-month cumulative incidences of vascularization development in the C-HHN group (16.9% and 55.8%, respectively) were compatible with those in a previous Japanese report (27.6% and 43.5%, respectively), which was based primarily on patients with HCV (25 out of 30 total patients were HCV-infected) [15]. The cumulative incidence of vascularization development in the BC-HHN group was significantly higher than that in the C-HHN group (Fig 3) and in the previous Japanese report.

More than half of the HHNs in the BC-HHN and C-HHN groups showed high-signal changes on T2-weighted images. This imaging finding is consistent with that of previous reports [18,19], in which a high baseline signal change in T2-weighted images was a strong risk factor for subsequent HHN vascularization development. Almost half of the HHNs in both groups showed restricted diffusion on diffusion-weighted imaging (DWI), which is consistent with results from a previous study [20] and strongly associated with vascularization development. In our study focusing on T2-weighted images or DWI, there were no significant differences between the two groups. Restricted diffusion on DWI tended to be a predictor in the univariate analysis (*p* = 0.121). However, by the multivariate analyses, the influence of signal on DWI or T2-weighted images did not affect the cumulative incidence of vascularization development.

Regarding the initial nodule size, HHN size >10 mm was reported as a predictor of vascularization development [15]. An initial HHN size difference between <10 mm and >12 mm was reported to have significantly different effects on the cumulative incidence of vascularization development [18]. In our study, HHN size >8 mm in BC-HHN group was not associated with vascularization development. According to the literature, the mean number of HHNs per patient that had vascularization development range from 1.29 to 1.85 [18,20]. In our study, the mean number of HHNs per patient was significantly higher in the BC-HHN group (1.53 vs. 1.07, *p* = 0.042). We noticed that HHNs in HBV and HCV co-infected patients tended to appear as smaller size and being more than one in number.

In 2013, Hyodo et al [21] reported that nodule volume doubling time of less than 542 days was associated with a high rate of vascularization development, based on the hepatitis C patients group. Based on our study, the nodule volume doubling time of C-HHN was concurrent with previous report. The nodule volume doubling time of BC-HHN was significant less than that of C-HHN. The nodule volume doubling time had significant positive correlation with vascularization development. However, in our study, the total number of nodules that had size changes and evaluated by nodule volume doubling time was small (31 nodules and 25 nodules, respectively). The impact of nodule volume doubling time on vascularization development is still unknown. Further prospective studies are warranted to elucidate this important issue.

Our study has certain limitations. First, a selection bias may exist because of the previously noted exclusion criteria. Second, the number BC-HHN and C-HHN was relatively small. Third, this was a retrospective study, so the follow-up period was not consistent among the study patients. Thus, an appropriate follow-up period for early recognition of vascularization development in the high-risk group could not be determined in this study.

## Conclusions

The most significant finding of the current study was the shorter interval to vascularization development in BC-HHNs, which were detected during the HBP on gadoxetic acid-enhanced MRI. In an area endemic for both HCV and HBV, such as Taiwan, it is important to monitor the vascularization development of cirrhotic precursor nodules in patients with HBV and HCV co-infections, especially male patients.

## Supporting information

S1 Text Dataset of BC-HHN and C-HHN. (XLS)

S1 Fig Approval number 2014-09-001BC. (PDF)
