# Supplementary material for: Interval to vascularization development in cirrhotic precursor nodules in patients with hepatitis B and C virus co-infections
Source: PLoS One. 2017 Jun 7;12(6):e0178841. doi: 10.1371/journal.pone.0178841 (PMC5462400; doi:10.1371/journal.pone.0178841)
Supplement: S1 Fig — (PDF) [file pone.0178841.s002.pdf]

臺 北 榮 民 總 醫 院
TAIPEI VETERANS GENERAL HOSPITAL
201 SHIH-PAI ROAD, SEC. 2
TAIPEI, TAIWAN 11217
REPUBLIC OF CHINA
TEL: (886)-2-2871-2121(30 LINES)

同 意 臨 床 試 驗 證 明 書

查本院放射線部 邱乃祈醫師主持之「B 型肝炎及 C 型肝炎同時感染病患，接受 Primovist 顯影劑磁振造影，呈現低訊號無血管性之肝臟結節是否易趨向高血管性，回顧性觀察研究」（本院 IRB 編號：2014-09-001BC）臨床試驗案，業經本院 103 年 09 月 10 日人體試驗委員會(二)審查通過，有效期限至 104 年 09 月 09 日，特此證明。

計畫主持人須於到期前2個月至6週（至少前6週）提出持續審查之申請，本案須經本院人體試驗委員會通過後，方可繼續執行。（凡需送衛生福利部審核之計畫案件，須取得衛生福利部審核同意函後方可開始執行）

臺北榮民總醫院
人體試驗委員會
主任委員
何善台

中 華 民 國 1 0 3 年 0 9 月 1 0 日

Sep 10, 2014

To Whom It May Concern:

RE: High risk of vascularization of hypointense hypovascular nodule in patients with hepatitis B virus/hepatitis C virus coinfection: Primovist MRI observation study
Principal Investigator: Nai-Chi Chiu, M.D.
Protocol No:
Version date of documents:
1. Protocol : V2, 2014/09/05
2. Synopsis: V1, 2014/08/04
3. Informed Consent Form: waived
4. Case Report Form: V2, 2014/09/05
VGHIRB No.: 2014-09-001BC

According to the written operating procedures, GCP, and the applicable regulatory requirements, this study project is approved by the Institutional Review Board of Taipei Veterans General Hospital. The board is organized under, and operates according to International Conference on Harmonisation (ICH) / WHO Good Clinical Practice (GCP) and the applicable laws and regulations.
This approval is valid for 1 year till Sep 09, 2015. The principal investigator is required to submit the application for extension 6 weeks before the expiration date. (If indicated by the regulations and laws, this project should be taken after the approval of Ministry of Health and Welfare, R.O.C.)

Shung-Tai Ho, M.D.
Chairman
Institutional Review Board
Taipei Veterans General Hospital
Taiwan, R.O.C.
